# *Leishmania major* Survival in Selective *Phlebotomus papatasi* Sand Fly Vector Requires a Specific *SCG*-Encoded Lipophosphoglycan Galactosylation Pattern

**DOI:** 10.1371/journal.ppat.1001185

**Published:** 2010-11-11

**Authors:** Deborah E. Dobson, Shaden Kamhawi, Phillip Lawyer, Salvatore J. Turco, Stephen M. Beverley, David L. Sacks

**Affiliations:** 1 Department of Molecular Microbiology, Washington University Medical School, St. Louis, Missouri, United States of America; 2 Laboratory of Parasitic Diseases, Intracellular Parasite Biology Section, National Institutes of Health, Bethesda, Maryland, United States of America; 3 Department of Molecular and Cellular Biochemistry, University of Kentucky Medical Center, Lexington, Kentucky, United States of America; Stanford University, United States of America

## Abstract

Phlebotomine sand flies that transmit the protozoan parasite *Leishmania* differ greatly in their ability to support different parasite species or strains in the laboratory: while some show considerable selectivity, others are more permissive. In “selective” sand flies, *Leishmania* binding and survival in the fly midgut typically depends upon the abundant promastigote surface adhesin lipophosphoglycan (LPG), which exhibits species- and strain-specific modifications of the dominant phosphoglycan (PG) repeat units. For the “selective” fly *Phlebotomus papatasi Ppap*J, side chain galactosyl-modifications (scGal) of PG repeats play key roles in parasite binding. We probed the specificity and properties of this scGal-LPG PAMP (Pathogen Associated Molecular Pattern) through studies of natural isolates exhibiting a wide range of galactosylation patterns, and of a panel of isogenic *L. major* engineered to express similar scGal-LPG diversity by transfection of *SCG*-encoded β1,3-galactosyltransferases with different activities. Surprisingly, both ‘poly-scGal’ and ‘null-scGal’ lines survived poorly relative to *Ppap*J-sympatric *L. major* FV1 and other ‘mono-scGal’ lines. However, survival of all lines was equivalent in *P. duboscqi*, which naturally transmit *L. major* strains bearing ‘null-scGal’-LPG PAMPs. We then asked whether scGal-LPG-mediated interactions were sufficient for *Ppap*J midgut survival by engineering *Leishmania donovani*, which normally express unsubstituted LPG, to express a ‘*Ppap*J-optimal’ scGal-LPG PAMP. Unexpectedly, these “*L. major* FV1-cloaked” *L. donovani-SCG* lines remained unable to survive within *Ppap*J flies. These studies establish that midgut survival of *L. major* in *Ppap*J flies is exquisitely sensitive to the scGal-LPG PAMP, requiring a specific ‘mono-scGal’ pattern. However, failure of ‘mono-scGal’ *L. donovani*-*SCG* lines to survive in selective *Ppap*J flies suggests a requirement for an additional, as yet unidentified *L. major*-specific parasite factor(s). The interplay of the LPG PAMP and additional factor(s) with sand fly midgut receptors may determine whether a given sand fly host is “selective” or “permissive”, with important consequences to both disease transmission and the natural co-evolution of sand flies and *Leishmania*.

## Author Summary

Phlebotomine sand flies are tiny blood-feeding insects that transmit *Leishmania* protozoan parasites, which cause diseases afflicting millions of people. The world-wide distribution of *Leishmania* is determined by the availability of transmission-competent vectors. In the laboratory, some vectors support many different *Leishmania*, while others are highly restricted. This is best exemplified by *P. papatasi*, which transmit only *L. major* despite a wide distribution in regions endemic for many *Leishmania* species. *P. papatasi* “selectivity” can be reproduced experimentally, and has been attributed to β1,3-linked galactose side chains decorating the abundant *L. major* surface lipophosphoglycan (LPG) adhesin, which mediate parasite attachment to the *P. papatasi* midgut to prevent elimination when the digested blood meal is excreted. As geographically diverse *L. major* display very different LPG galactosylation patterns (n = 0 - 8 βGals/side chain), we explored the consequences of this pattern diversity to survival in *P. papatasi*. Using natural isolates and *L. major* lines engineered to express a wide range of LPG galactosylation patterns, we showed *L. major* survival in *P. papatasi Ppap*J flies was optimized by expression of highly modified ‘mono-galactosylated’ LPG and extremely sensitive to LPG side chain length. Surprisingly, *L. donovani* lines engineered to express a “*Ppap*J-optimal” LPG mono-galactosylation pattern did not survive in *Ppap*J flies, suggesting that additional interactions are required. These studies reveal the fine specificity of *Leishmania* - sand fly interactions, and the nature of species- and strain-specific parasite molecules that have co-evolved to take advantage of midgut receptors specific to available sand fly vectors.

## Introduction

*Leishmania* are protozoan parasites that cause a spectrum of human diseases that range from self-healing cutaneous lesions to potentially fatal visceral forms. Leishmaniasis is re-emerging as a significant world health problem, with approximately 12 million people presently infected and 2 million new cases diagnosed each year (www.who.int/leishmaniasis/burden).

The world-wide distribution of different *Leishmania* is determined by the availability of transmission-competent sand fly vectors. When a sand fly bites an infected vertebrate host, *Leishmania* amastigotes residing within macrophages and other cell types are taken up in the blood meal which is surrounded by a midgut peritrophic matrix that lasts for several days. During this time amastigotes differentiate into motile, replicating promastigote forms which reside in the extracellular lumen of the sand fly alimentary tract (rev. in [1], [2], [3]). Barriers to *Leishmania* development in this compartment include the chitin-containing peritrophic matrix which completely encases the blood meal, and the many hydrolytic enzymes and anti-microbial molecules secreted into the gut lumen (rev. in [2], [3], [4], [5]). Eventually the remnants of the digested blood meal are excreted by the sand fly, and this is a crucial juncture for *Leishmania* promastigotes. In transmission-competent sand flies, parasites attach to the midgut epithelium and go on to establish a stable infection; in a transmission-refractory vector, unattached parasites are expelled when the sand fly defecates (rev. in [2], [3], [4]). As the sand fly prepares to feed again, promastigotes transition through several forms that culminate in infectious metacyclic parasites which express a modified surface that cannot bind to the midgut epithelium (rev. in [1], [3], [4], [6]). Thus, a key step in *Leishmania* transmission is stage-specific midgut attachment which allows *Leishmania* development to proceed.

Two distinct mechanisms for regulating *Leishmania* attachment to sand fly midgut epithelium have been identified to date (rev. in [1], [6], [7], [8]). One mechanism, utilized in “selective” *Phlebotomus papatasi* sand flies that support the complete development of only a single *Leishmania* species, involves a sand fly midgut epithelium receptor that binds the parasite lipophosphoglycan (LPG) adhesin. LPG is an abundant glycolipid that covers the entire surface, including the flagellum, of all *Leishmania* promastigote stages [9]. The basic LPG structure is highly conserved in all *Leishmania* species, consisting of a glycosyl-phosphatidyl-inositol lipid anchor to which is attached a long polymer of 10–30 phosphoglycan (PG) repeating units (6Galβ1,4-Manα1-PO_4_), and terminated by a small neutral oligosaccharide cap (rev. in [10], [11]). The PG repeating units are often modified by strain-, species-, and developmental stage-specific modifications that have been implicated in the midgut attachment and release of several *Leishmania* species in their respective natural vectors [12], [13], [14], [15]. A second, LPG-independent sand fly midgut binding mechanism was recently identified using LPG-deficient *Leishmania* and several “permissive” sand flies that support the development of a broad range of *Leishmania* species in the laboratory [2], [7], [8], [16]. While the precise binding modality is uncertain, the involvement of vector glycans has been suggested [8], [16]. *Leishmania* phosphoglycans and/or other *LPG2*-dependent molecule(s) are also required for parasite survival in “permissive” sand flies [8], [17]. Thus sugars, in the form of surface glycoconjugates, are key players in the productive interactions between *Leishmania* and sand flies that are necessary for disease transmission. This is in agreement with the general principle that important surface interactions between many microbes and their hosts involve complex glycoconjugates binding to receptors (rev. in [18]).

In this study we focused on the interactions between *Leishmania major* promastigotes and *Phlebotomus papatasi* sand flies. *Phlebotomus papatasi* is a “selective” vector which, despite its wide distribution in regions endemic for transmission of several *Leishmania* species, transmits only *Leishmania major* in nature and in the laboratory (rev. in [1], [2], [3], [4], [6]. In this vector, specificity is controlled by a stage-specific modification in the LPG adhesin [6], [12]. Midgut attachment is mediated by modified PG repeats bearing side chain β1,3 galactosyl residues (scGal), which form the ligand recognized by the midgut LPG receptor PpGalec identified in Jordan Valley strain *P. papatasi* (*Ppap*J) sand flies [19]. As *L. major* procyclics develop into infectious metacyclic forms, procyclic form LPG is shed and replaced by metacyclic form LPG, which has increased numbers of PG repeats and scGal residues masked by the addition of terminal arabinose “caps”; these modifications block binding to PpGalec receptors [19] and facilitate detachment from the midgut [19], [20]. Laboratory infections established the requirement for scGal-LPG in *Ppap*J midgut survival: *L. major* mutants or *Leishmania* species expressing scGal-deficient LPG, or lacking LPG entirely, could not establish stable *Ppap*J infections [12], [16], [21], [22], [23], [24] and bound poorly to isolated *Ppap*J midguts and recombinant PpGalec receptors *in vitro* [19].

Notably, geographically diverse *L. major* strains express very different LPG side chain galactosylation patterns [which we refer to hereafter as scGal-LPG PAMPs (Pathogen Associated Molecular Patterns)], showing a Southwest-to-Northeast cline across its range from ‘null-scGal’ to ‘poly-scGal’ LPG PAMPs ([23], [25], [26], [27]; Cardoso *et al.*, in preparation}. For example, Senegalese strain SD procyclic LPG has such low levels of single βGal modifications that it is effectively unmodified [‘null-scGal’ LPG PAMP; [23] and this report}. In contrast, Israeli strain FV1 procyclic LPG is highly modified with primarily single βGal residues (‘mono-scGal’ LPG PAMP; [27]), and Central Asian strain LV39 clone 5 (LV39c5) procyclic LPG is highly modified by long polymers of up to 8 βGal residues (‘poly-scGal’ LPG PAMP; [28], [29]). Amongst these natural *L. major* strains, FV1 is sympatric with the “selective” *P. papatasi Ppap*J sand fly, while SD parasites are sympatric with *P. duboscqi*, a closely related sibling species of *P. papatasi* (rev. in [3]).

Previously, we hypothesized that different scGal-LPG PAMPs resulted from the combined activity of the seven telomeric *SCG* (*Side Chain Galactose*) gene family members, which encode PG-side chain-β1,3-galactosyltransferases. *SCG*s exhibit different activities, combining to varying extents ‘initiating’ activities able to attach the first βGal residue to the basic PG repeat, and ‘elongating’ activities able to add additional βGal residues to the initiated βGal side chain ([30], [31]; Dobson *et al.*, in preparation). In this work, we made use of this suite of diverse *SCG* activities to engineer isogenic parasites bearing defined scGal-LPG PAMPs. Using both naturally-occurring *L. major* strains and SD-*SCG* transfectant lines, we show that a specific scGal-LPG PAMP is optimal for long-term parasite survival in selective *Ppap*J sand flies, which preferred highly substituted scGal-LPG PAMPs bearing mono-galactosyl chains, neither “too short” nor “too long”. The “*Ppap*J-optimal” scGal-LPG PAMP was not sufficient, however, to enhance survival of *L. donovani*-*SCG* transfectants in *Ppap*J sand flies. These findings lead us to propose a two-component model for long-term *Leishmania* survival in “selective” *Ppap*J sand flies: 1) a specific scGal-LPG PAMP recognized by PpGalec midgut receptors and 2) an as yet unidentified *L. major* species-specific factor(s).

## Methods

### *Leishmania* strains and transfections

*Leishmania major* strain Friedlin V1 (FV1) is a clonal derivative of the Friedlin line (MHOM/IL/80/Friedlin), *L. major* strain LV39 clone 5 (LV39c5) is a clonal derivative of the LV39 line (RHO/SU/59/P), *L. major* strain SD 75.1 (SD) is a clonal derivative of the NIH/SD line (MHOM/SN/74/SD), *L. donovani* Sudanese strain 1S-2D clone Ld4 (*Ld*) is a clonal derivative (MHOM/SD/00/1S-2D), and *L. mexicana* strain M379 is a clonal derivative (MYNC/BZ/62/M379). All wild type (WT) lines showed good infectivity in animal models and in their natural sand fly vectors [24], [32], [33], [34]. Cells were grown in complete M199 medium containing 10% heat-inactivated fetal bovine serum, penicillin (50 units/ml), streptomycin (50 µg/ml), HEPES pH 7.4 (40.5 mM), adenine (0.1 mM), biotin (0.0001%), biopterin (2 µg/ml), and hemin (0.0005%), at 25°C as described [35]. Procyclic promastigotes were harvested from logarithmically growing cultures.

Promastigotes were transfected by electroporation, using a low voltage [35] or high voltage [36] protocol. Clonal lines were obtained by plating on semisolid M199 media containing the appropriate selective drug concentration: 50 µg/ml hygromycin B (HYG), 20 µg/ml phleomycin (PHLEO), 15 µg/ml G418 (NEO), or 100 µg/ml nourseothricin (SAT).

### Molecular constructs and transfectants

*L. major* strain FV1 was the source of all *SCG* genes used in this study. Episomal expression constructs used here include the cosmid vector cLHYG (strain B890; [37]), pXK(*NEO*)-*SCG2* (B3900; [30]), *SCG3* cosmid B3979 [30], and pXG(*NEO*)-LM*SAP1* (B3092; [34]). The integrating *SCG* open reading frame (ORF) constructs pIR1SAT-*SCG1* (B5097), pIR1SAT-*SCG3* (B5101), pIR1SAT-*SCG4* (B5103), and pIR1SAT-*SCG5* (B5170) were created as follows: *SCG* ORFs liberated by BamHI digestion of appropriate pXG(*PHLEO*)-*SCG* ORF constructs (Dobson *et al.*, in preparation) were ligated into the *Bgl*II expression site of pIR1SAT (B3541; [36]). Each pIR1SAT-*SCG* construct was digested with SwaI restriction enzyme, dephosphorylated with calf alkalline phosphatase, and gel-purified to yield linear *SSU::IR1SAT-SCG* ORF targeting fragments for integration into the ribosomal RNA small subunit (*SSU*) locus by homologous recombination during transfection [36]. Integrated *SCG* transfectants are referred to by the gene name and location, i.e. SD-*SSU:SCG3* has the FV1 strain *SCG3* ORF (*SSU::IR1satSCG3*) integrated into the SD ribosomal *SSU* locus.

We used three *Ld*-transfectant lines developed previously [30]: a ‘null-scGal’ LPG PAMP line devoid of any LPG side chain modification, transfected with the episomal cosmid vector cLHYG (*Ld*-*vector*); and two different lines exhibiting ‘mono-scGal’LPG PAMPs, one transfected with *SCG3* cosmid B3979 (*Ld*-c*SCG3*), and a second transfected with the *SCG2* ORF expression construct pXK-*SCG2* (*Ld-pSCG2*).

### Purification and analysis of LPG

LPG was prepared from exponentially-growing promastigotes as described [38]. To assess side chain modifications, phosphoglycan repeats were depolymerized using mild acid hydrolysis, dephosphorylated using *E. coli* alkaline phosphatase, covalently labeled with 1-aminopyrene-3,6,8-trisulfonate, and analyzed by Dionex HPLC chromatography [14] or capillary electrophoresis [39], comparing migration distances with oligomeric glucose standards.

### Analysis of secreted acid phosphatase (SAP) levels

Procyclic promastigotes (5×10^5^/ml) were grown in complete M199 medium to a final density of 1×10^7^/ml. Culture supernatants were collected and centrifuged for 10 min at 2500 rpm in a Sorvall RT7000 centrifuge to remove cells and debris. 10 microliter samples of clarified culture supernatant were electrophoresed on a non-denaturing polyacrylamide gel and the gel then stained for SAP enzyme activity using α-naphthyl acid phosphate plus Fast Garnet GBC as described [40], [41]. SAP was quantitated using the AlphaImager version 5.5 gel documentation system spot densitometry program (Alpha Innotech, San Leandro, CA).

### Sand fly infection and dissection

Sand fly colonies were reared at the Division of Entomology, Walter Reed Army Institute of Medical Research and at the Laboratory of Parasitic Diseases, National Institute of Allergy and Infectious Diseases, NIH. The following species were used in this study: *Phlebotomus papatasi* from colonies originating from the Jordan Valley (*Ppap*J), *Phlebotomus duboscqi* from colonies originating from Mali (*Pdub*M), and *Phlebotomus argentipes* from colonies originating from India (*Parg*IN).

Female 3- to 5-day-old sand flies were fed through a chick skin membrane using a feeding device containing a mixture of heparin-treated mouse blood and logarithmic phase promastigotes, as described [24]. The concentration of promastigotes used varied depending on the experiment, from 1×10^6^ to 20×10^6^ parasites/ml. Blood-engorged sand flies were separated and maintained at 28 C with 30% sucrose (v/v). At various times after feeding, flies were anesthetized, their midguts dissected and homogenized, and the number of released midgut promastigotes counted using a hemocytometer as described [24].

### Statistical analyses

Parasite numbers in the midguts of infected flies after blood meal excretion do not follow a Gaussian distribution. This is likely the result of flies within groups having either completely lost their infections or retained parasites that grow exponentially prior to the time of dissection. Therefore, data sets were compared using a nonparametric Mann Whitney test. Mann Whitney calculations were done using Prism 4 (Graphpad Software, Inc. San Diego, CA).

## Results

### Three natural *L. major* strains show varying patterns of LPG side chain galactosylation

LPGs from three geographically distinct *L. major* strain procyclic promastigotes were purified, subjected to mild acid hydrolysis and dephosphorylation, and isolated PG repeat structures assessed by capillary electrophoresis (Methods, Table S1). Side-chain galactosylation can be characterized by two parameters: the fraction of PG repeats that were modified, and the number of βGal residues attached. In these studies we found two general patterns of LPG side chain galactosylation: one in which little or no βGal was added; and a second in which 50–90% of the PG repeats were modified, with varying numbers of βGal residues. From these data we found it useful to calculate a single parameter for comparisons amongst lines, the “average scGal chain length”, obtained by multiplying the fraction of modified PG repeats times the average number of βGal residues added per modified repeat (Tables 1, S1).

**Table 1:** Effect of LPG galactosylation pattern on *Leishmania* survival in “selective” *Phlebotomus papatasi PpapJ* sand flies.

*Leishmania* line^a^	LPG-scGal modification frequency^b^	avg. scGal chain length^c^	scGal-LPG PAMP^d^	*Ppap*J survival post-blood meal expulsion		
				% infected flies^e^	relative parasites/midgut^f^	average relative survival^g^
WT FV1	71%	0.8	mono	96±5	100	96±5
WT LV39c5	93%	3.1	poly	61±8	32±8	20±7
WT SD	2%	0.02	null	38*	41*	16*
SD-SSU:SCG5	2%	0.02	null	61±1	14±5	8±3
SD-cSCG3	68%	0.9	mono	90±4	100±37	89±30
SD-SSU:SCG3	86%	1.3	mono	88±12	91±48	75±31
SD-SSU:SCG1	56%	1.9	oligo	46±9	36±6	16±1
SD-SSU:SCG4	54%	3.1	poly	49±7	6±1	3±1
Ld-vector	0%	0	null	19±19	4±4	1±1
Ld-cSCG3	65%	0.7	mono	8*	1*^,^∧	0.2*^,^∧
Ld-pSCG2	62%	1.1	mono	26±26	4±4	2+2

a The *L. major* (FV1, LV39c5, SD) and *L. donovani* (*Ld*) wild-type (“WT”) and transfectant lines used in experimental *Ppap*J laboratory infections are described in the text, with supporting data in Tables S1 to S3 and Methods.

b “LPG-scGal modification frequency” is the percentage of modified PG repeats bearing terminal βGal side chains present in purified procyclic promastigote LPG samples.

c The average length of βGal side chains in purified procyclic promastigote LPG, or “avg. scGal chain length”, was calculated by multiplying “LPG-scGal modification frequency” × “mean scGal chain length”, using data in Table S1.

d LPG galactosylation patterns, or “scGal-LPG PAMPs”, were classified by the average length of LPG βGal side chains (column 3): ‘null’, <0.1 βGal; ‘mono’, 0.7–1.3 βGals; ‘oligo’, 1.9 βGals; ‘poly’, ≥3 βGals.

e “% infected flies” is the average percentage (±SEM) of *Leishmania*-infected *Ppap*J flies post-blood meal expulsion, calculated from data in Figs. 1, 2, 4 and Tables S2, S3. Most lines were examined in two independent infections, except for WT FV1 (6 infections), and WT SD and *Ld*-c*SCG3* (1 experiment each, “*”).

f “Relative parasites/midgut” is the average number (± SEM) of parasites per midgut post-blood meal expulsion calculated relative to WT FV1  = 100, using data in Figs. 1, 2, 4, and Tables S2, S3. The average of two or more independent experiments is shown, except for WT SD and *Ld*-c*SCG3* (1 experiment each, “*”). ∧Since WT FV1 was not included in this experiment, and *P. argentipes Parg*IN is the natural vector for *Ld* parasites, the number of *Ld*-c*SCG3* promastigotes was calculated relative to *Ld-vector*-infected *Parg*IN flies.

g The “average relative survival” of parasites post blood-meal expulsion was calculated by multiplying “% infected flies” × “relative parasites/midgut” (column 7× column 8). The average (± SEM) of two independent experiments is shown, except for WT FV1 (6 experiments) and SD and *Ld*-c*SCG3* (1 experiment each, “*”). ∧Since WT FV1 was not included in this experiment, and *Parg*IN is the natural vector for *Ld* parasites, survival is relative to control *Ld-vector*-infected *Parg*IN flies.

Senegalese strain SD LPG was mostly unmodified (0.02 avg. scGal chain length), consistent with prior studies using specific antisera and lectins suggesting that SD LPG was largely unmodified [23]. In contrast, Israeli strain FV1 LPG was extensively modified with predominantly single βGal residues (0.8 avg. scGal chain length). Central Asian strain LV39c5 LPG was also highly modified, but with longer polymers of up to 8 βGal residues (3.1 avg. scGal chain length). We refer to these three prototypic LPG galactosylation patterns as ‘null-scGal’, ‘mono-scGal’, and ‘poly-scGal’ LPG PAMPs (Pathogen-Associated Molecular Patterns), respectively. The results with FV1 and LV39c5 confirmed and extended previous studies [27], [28], [29], and were undertaken to guard against changes in LPG side chain composition occurring during laboratory propagation, as described previously [25], [42].

### *L. major* survival in selective *Phlebotomus papatasi Ppap*J sand flies requires a specific scGal-LPG PAMP

*Ppap*J sand flies were fed on the indicated *L. major*-infective mouse blood and midgut infections were assessed 48 hr later, a time when parasites remain within the blood meal encased by the peritrophic membrane (Fig. 1A, “+ blood, d2”). At this time all three *L. major* strains showed high parasite numbers in most flies examined (>33,000 parasites/midgut), with the highest numbers observed in flies infected with the SD strain, likely reflecting the faster generation time of this strain. Thus differences in the scGal-LPG PAMPs did not affect the early survival and growth of *L. major* promastigotes, as expected since even LPG-deficient parasites survive normally in sand flies during this interval [16], [22], [24].

**Figure 1:**
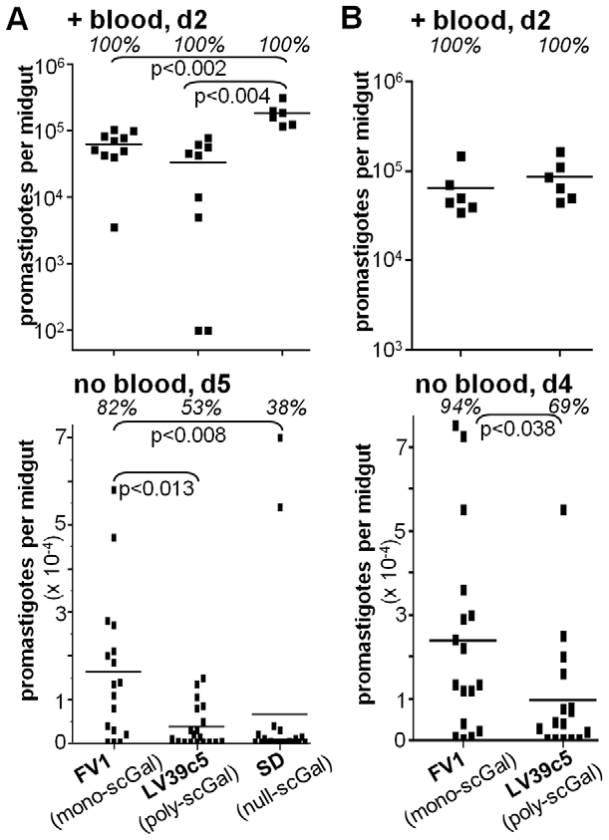
Galactosylated LPG does not ensure survival of *L. major* promastigotes in Jordan Valley strain *P. papatasi Ppap*J sand flies. Female *Ppap*J sand flies were membrane fed on infective mouse blood containing the indicated *L. major* strain (LPG galactosylation pattern in parentheses) at concentrations of 4×10^6^ (panel A) or 8×10^6^ (panel B) per ml. At the indicated day (“d”) after feeding, midguts were dissected and the number of viable promastigotes determined by counting under a hemocytometer. “+ blood” denotes midguts that retained the blood meal, and “no blood” denotes midguts that had no detectable blood as a result of the digested blood meal having been expelled. Each symbol represents the number of parasites in a single sand fly midgut, and each bar represents the mean number of parasites for each group. The percentages of infected flies in each group are shown in italics. P values shown were calculated for the indicated pairs of infected flies. Results from two independent experiments (panels A,B) are shown.

By day 5 post-feeding, the sand fly peritrophic matrix disintegrates and the remains of the digested blood meal are expelled. At this time there were clear differences amongst the *L. major* strains in their ability to survive (Fig. 1A, “no blood, d5”). In agreement with previous studies [22], [24], ‘mono-scGal’ FV1 persisted in most *Ppap*J flies at high levels (82% flies infected, 16200±16600 parasites/midgut). In contrast, ‘poly-scGal’ LV39c5 survived poorly (53% flies infected, 3860±4840 parasites/midgut; p<0.013), as did ‘null-scGal’ SD, with the exception of two strongly-infected outliers (38% flies infected, 6660±18600 parasites/midgut; p<0.005). The poor survival of ‘null-scGal’ SD was expected, as un-galactosylated LPG cannot bind to midgut PpGalec receptors [19], resulting in unattached parasites being excreted with the digested blood meal remnants [21], [24]. However, the poor *Ppap*J survival of ‘poly-scGal’ LV39c5 (Fig. 1A,B; [24]) suggested that a specific scGal-LPG PAMP, rather than simply the presence of galactosylated LPG, controls *L. major* promastigote survival in *Ppap*J midguts following blood meal expulsion.

### Generation of isogenic *L. major* parasites bearing a range of scGal-LPG PAMPs

Since the three *L. major* strains studied here show an average nucleotide sequence divergence of 0.15% [43], comparable to that amongst many *L. major* strains, molecular differences other than scGal-LPG PAMPs were potentially responsible for the survival differences we observed in selective *Ppap*J sand fly infections. To generate different scGal-LPG PAMPs in an isogenic scGal-deficient LPG background, we introduced into the SD line a series of constructs expressing members of the previously characterized *SCG* family of telomeric phosphoglycan-side chain-(β1,3)galactosyltransferases (PG-scβGalTs) [30], [31] Critical to these studies is the fact that *SCG*-encoded PG-scβGalTs have different enzymatic properties mediating the addition of different numbers of scGal residues, ranging from 0 to 12 ([30], [31]; Dobson *et al*., in preparation). Thus, SD promastigotes were transfected with different *SCG* constructs, using either the episomal pXG-type vector which expresses passenger ORFs at moderate levels [37], episomal cosmids identified previously bearing *SCG* genes [30], or the integrating pIR1SAT vector which expresses passenger ORFs at high levels following integration into the ribosomal RNA small subunit (*SSU*) locus [36]. LPGs were purified from SD transfectants and LPG galactosylation patterns determined as described above (Methods); from these studies we chose a key set of SD-*SCG* lines exhibiting a range of scGal-LPG PAMPs (Tables 1, S1) briefly summarized here.

SD transfectants bearing an integrated catalytically inactive *SCG5* ORF (SD-*SSU:SCG5*) synthesized scGal-deficient LPG indistinguishable from the parental WT SD line (‘null-scGal’ LPG PAMP; 0.02 avg. scGal chain length). Two SD transfectants expressed ‘mono-scGal’ LPG PAMPs: SD-c*SCG3* (0.9 avg. scGal chain length), containing the episomal *SCG3* cosmid B3979; and SD-*SSU:SCG3* (1.3 avg. scGal chain length), containing an integrated *SCG3* ORF (*SSU::IR1SAT*-*SCG3*). SD-*SSU:SCG4* transfectants bearing an integrated *SCG4* ORF (*SSU::IR1SAT-SCG4*) synthesized a ‘poly-scGal’ LPG PAMP (3.1 avg. scGal chain length). A novel ‘oligo-scGal’ LPG PAMP (1.9 avg. scGal chain length) was synthesized by SD-*SSU:SCG1*, which bears an integrated *SCG1* ORF (*SSU::IR1sat*-*SCG1*). Together these SD-transfectant scGal-LPG PAMPs spanned the natural range of *L. major* LPG side chain variation as well as providing new LPG galactosylation patterns for study.

To confirm that SD transfectants had not experienced a general non-specific loss of “sand fly virulence” during their generation and propagation in the laboratory, we examined their survival in two independent infections involving *Phlebotomus duboscqi Pdub*M sand flies originating from Mali (Fig. S1 A,B). *P. duboscqi* is a sibling species of *P. papatasi*, and *Pdub*M flies are able to support the full development of WT SD in the laboratory [23]. Although *L. major* survival in *P. duboscqi* is LPG-dependent, it is not strongly affected by scGal-LPG PAMPs since various ‘null-scGal’, ‘mono-scGal’, or ‘poly-scGal’ *L. major* strains have been shown to survive expulsion of the digested blood meal [8], [17], [22], [44]. Female *Pdub*M sand flies were allowed to feed on the indicated *L. major*-infective mouse blood containing ‘null-scGal’ (WT SD, SD-*SSU:SCG5*), ‘mono-scGal’ (WT FV1, SD-*SSU:SCG3*), ‘oligo-scGal’ (SD-*SSU:SCG1*), or ‘poly-scGal’ (SD-*SSU:SCG4*) promastigotes. As expected, all *Pdub*M flies were successfully infected with high numbers of parasites at early time points (Fig. S1, “+ blood” panels). Following expulsion of the digested blood meal, *Pdub*M flies infected with all *L. major* lines retained high numbers of midgut parasites (Fig. S1 “no blood” panels) and each line went on to establish fully mature infections in the *Pdub*M anterior midgut by day 12 post-feeding (data not shown). These data argue against a general non-specific loss in the ability of SD-*SCG* transfectants to survive in the phlebotomine sand fly midgut environment.

### Effect of scGal-LPG PAMPs on SD transfectant survival in “selective” *Ppap*J sand flies

**Figure 2 ppat-1001185-g002:**
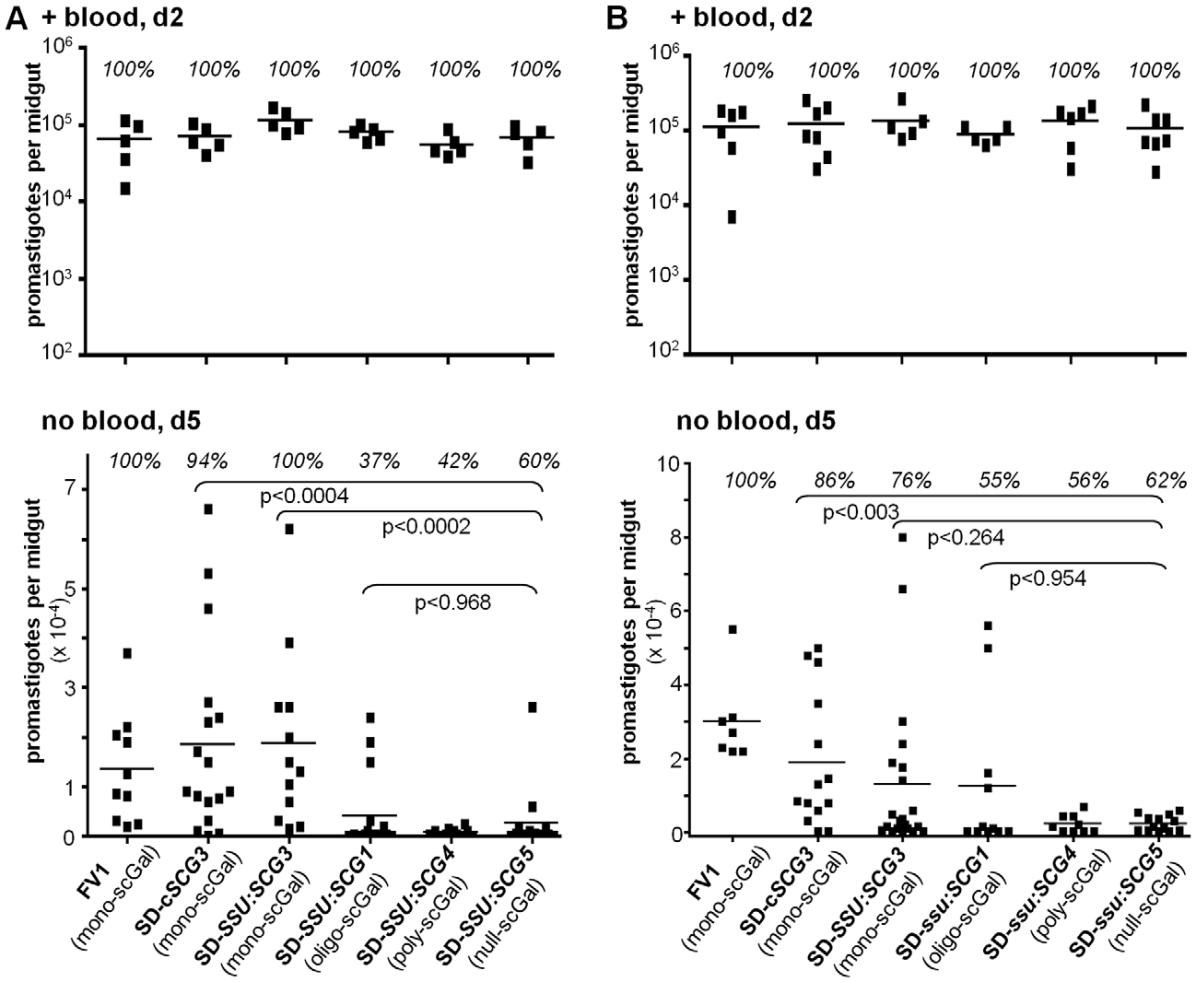
Survival of *L. major* SD-*SCG* transfectants in *Ppap*J sand flies is dependent on expression of specific scGal-LPG PAMPs. Female *Ppap*J flies were membrane fed on the indicated *L. major*-infective mouse blood and the number of viable parasites per midgut determined on the indicated day post-feeding as described in Fig. 1. SD-transfectant lines are described in the text, with additional data in Table S1. Infective mouse blood contained 5×10^6^ (panel A) or 10×10^6^ (panel B) parasites per ml. Results from two independent experiments (panels A,B) are shown.

*Ppap*J flies were allowed to feed on the indicated *L. major*-infective mouse blood containing SD transfectants expressing different scGal-LPG PAMPs. The results from two independent experiments are shown (Fig. 2A,B). At early times post-infection when the midgut blood meal was retained, all SD transfectants behaved similarly: 100% of *Ppap*J flies were infected with high numbers of promastigotes, similar to control ‘mono-scGal’ WT FV1 infections (Fig. 2A,B, “+ blood, d2”). However, we observed clear differences in *Ppap*J midgut survival amongst SD lines expressing different scGal-LPG PAMPs after the digested blood meal had passed out of the midgut (Fig. 2A,B, “no blood, d5”; Tables 1, S2).

First, and as expected, SD-*SSU:SCG5* transgenic parasites expressing a ‘null-scGal’ LPG PAMP survived poorly following blood meal excretion, with a 81–92% decrease in mean parasite numbers relative to control FV1-infected flies (p<0.0005), and 38–40% of *Ppap*J flies having lost their infections (Fig. 2A,B, “no blood, d5”).

Second, SD transfectants expressing ‘mono-scGal’ LPG PAMPs (SD-c*SCG3*, SD-*SSU:SCG3*) generally survived well post-blood meal expulsion (Fig. 2A,B, “no blood, d5”). Most flies remained infected, and mean SD-c*SCG3* and SD-*SSU:SCG3* parasite numbers (18600±4732, 18900±4900 and 18800±5100, 13200±4800 parasites/midgut, respectively) were not significantly different from control WT FV1 infections (13500±3500, 30000±4400 parasites/midgut). By contrast, ‘mono-scGal’ SD-c*SCG3* survival was significantly enhanced relative to ‘null-scGal’ SD-*SSU:SCG5* (2560±1720, p<0.0004 and 2420±600 parasites/midgut, p<0.003; Fig. 2A,B). Although ‘mono-scGal’ SD-*SSU:SCG3* survival was also enhanced relative to ‘null-scGal’ SD-*SSU:SCG5*, this difference reached significance in only one experiment (p<0.0002 and p<0.264; Fig. 2A,B).

Third, SD-*SSU:SCG1* transfectants expressing a novel ‘oligo-scGal’ LPG PAMP survived poorly. Only 37–55% of SD-*SSU:SCG1* flies remained infected post-blood meal expulsion and parasite levels (4120±1940, 12500±6300 parasites/midgut) were significantly reduced relative to control WT FV1-infected flies (p<0.018 and p<0.06; Fig. 2A,B). In fact, ‘oligo-scGal’ SD-*SSU:SCG1* survival was not significantly better than observed for ‘null-scGal’ SD-*SSU:SCG5* (p<0.968 and p<0.954; Fig. 2A,B).

Lastly, and consistent with the results from natural isolates, SD-*SSU:SCG4* transfectants expressing a ‘poly-scGal’ LPG PAMP survived poorly. Only 42–56% of SD-*SSU:SCG4* flies remained infected and parasite levels (683±215, 2267±831 parasites/midgut) were significantly decreased, 92–95% relative to control WT FV1 infections (p<0.0007 and p<0.0001, Fig. 2A,B). Thus ‘poly-scGal’ SD-*SSU:SCG4 Ppap*J survival was not significantly better than ‘null-scGal’ SD-*SSU:SCG5* parasites.

**Figure 3:**
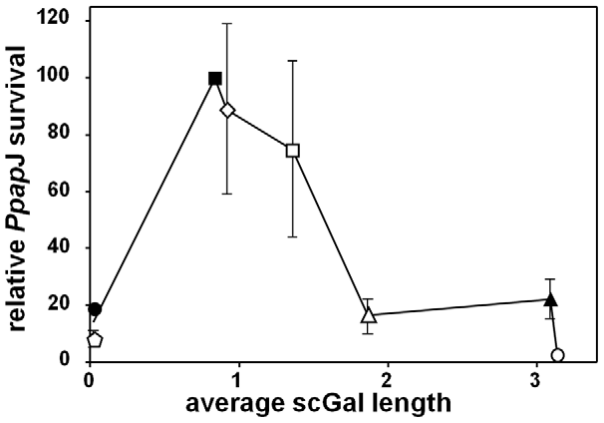
Relationship between *L. major* scGal-LPG PAMPs and *Ppap*J midgut survival post-blood meal expulsion. Relative survival of *L. major* promastigotes in infected *Ppap*J sand flies which have expelled their digested blood meal is plotted as a function of the average LPG scGal length, using data in Table 1. The average (±SEM) of two or more independent experiments is shown for FV1 (▪), LV39c5 (▴), SD-*SSU:SCG5* (open pentagon), SD-c*SCG3*(⋄), SD-*SSU:SCG3* (□), SD-*SSU:SCG1* (▵), and SD-*SSU:SCG4* (○). We include data here for SD (•) from a single experiment, which is consistent with previous studies [21], [23].

These findings are summarized in Fig. 3, showing the relationship between relative *Ppap*J survival post-blood meal expulsion and the average scGal chain length in purified procyclic promastigote LPG. Isogenic SD-*SCG* transfectants whose LPG closely approximates the ‘mono-scGal’ LPG PAMP of the WT FV1 line (i.e. SD-c*SCG3*, SD-*SSU:SCG3*) clearly survived well in *Ppap*J sand flies. In contrast, isogenic SD transfectants expressing either scGal-deficient LPG (‘null-scGal’ SD-*SSU:SCG5*) or LPG with longer side chain polymers (‘oligo-scGal’ SD-*SSU:SCG1*, ‘poly-scGal’ SD-*SSU:SCG4*) survived poorly in *Ppap*J flies, mirroring infection outcomes with naturally-occurring *L. major* strains SD or LV39c5 (‘null-scGal’ or ‘poly-scGal’ LPG PAMPs, respectively). Together, these data firmly implicate the scGal-LPG PAMP causally in controlling the ability of *Ppap*J flies to support *L. major* midgut survival post-blood meal expulsion.

### Are “*Ppap*J-optimal” scGal-LPG PAMPs sufficient to enhance *Ppap*J survival of a different *Leishmania* species?

The studies above established that a ‘mono-scGal’ LPG PAMP was necessary for *L. major* survival in selective *Ppap*J sand flies, following blood meal expulsion. We next asked whether this scGal-LPG PAMP would be sufficient, by examining its effect on the *Ppap*J survival of a different *Leishmania* species. We chose *L. donovani* Sudanese strain 1S-2D (*Ld*) since these parasites possess unmodified LPG (‘null-scGal’ LPG PAMP; [11], [45]) and have been shown to survive poorly in *Ppap*J sand flies [12], [24]. We used three *Ld*-transfectant lines developed previously [30]: a ‘null-scGal’ line devoid of any side chain sugars (*Ld*-*vector*, 0 avg. scGal length) and two different lines exhibiting ‘mono-scGal’ LPG PAMPs, *Ld*-c*SCG3* and Ld-p*SCG2* (0.7 and 1.1 avg. scGal chain length, respectively; Tables 1, S1).

**Figure 4:**
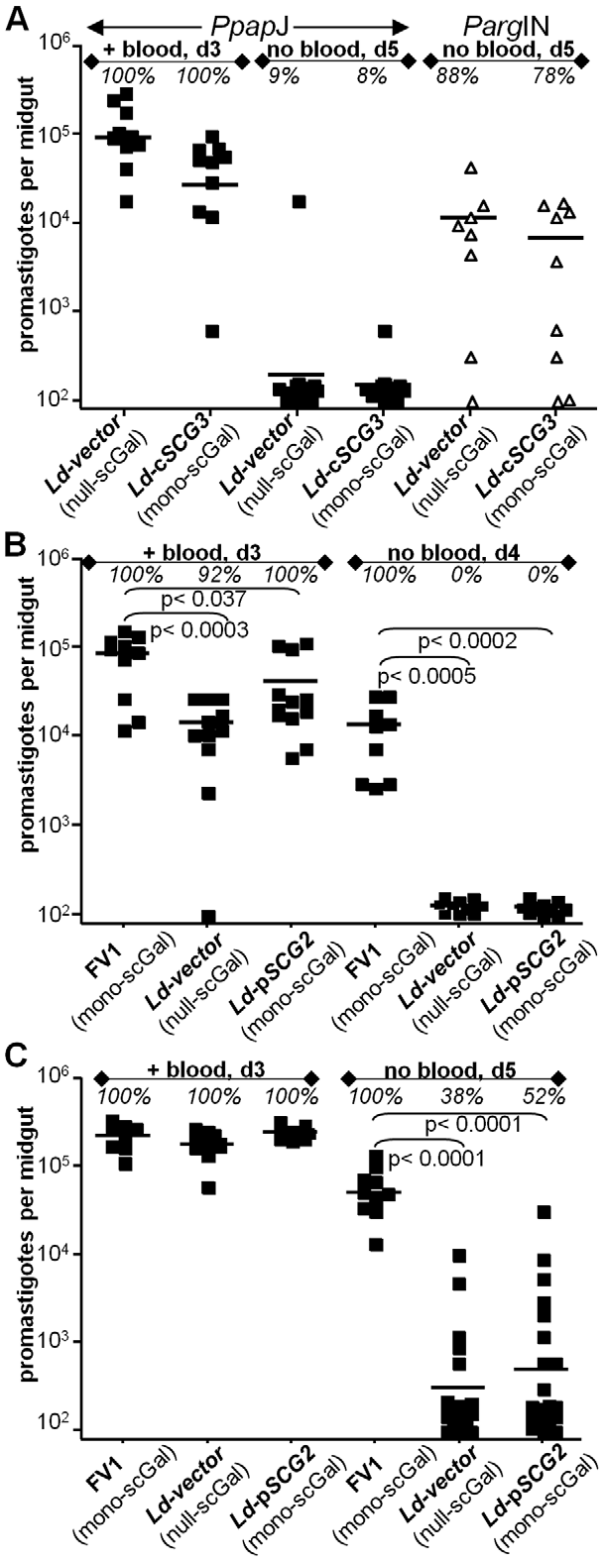
Expression of “*Ppap*J-optimal” scGal-LPG PAMPs in *L. donovani-SCG* transfectants does not improve survival in *Ppap*J sand flies. Female sand flies were membrane fed on the indicated *Leishmania*-infective mouse blood and the number of viable parasites per midgut determined on the indicated day post-feeding as described in Fig. 1. *Ld* transfectant lines are described in the text, with additional data in Table S1. In panel A, *P. papatasi Ppap*J (“*Ppap*J”) and *P. argentipes Parg*IN (“*Parg*IN”) flies fed on infective blood containing 10×10^6^ parasites per ml. In separate experiments, *P. papatasi Ppap*J flies fed on infective blood containing 5×10^6^ (panel B) or 20×10^6^ (panel C) parasites per ml.

When *Ppap*J sand flies were fed on *L. donovani*-infective mouse blood containing ‘null-scGal’ *Ld-vector* or ‘mono-scGal’ *Ld-cSCG3* promastigotes, all flies were successfully infected with comparably high numbers of parasites when examined at a time when the midgut blood meal was present (Fig. 4A, *Ppap*J “+ blood, d3” panel). Thus, these parasites were able to survive well in the initial steps of sand fly infection. However, following expulsion of the blood meal at day 5 post-feeding, parasites from both of these lines were completely lost in >90% of *Ppap*J flies, and those flies retaining infections had very low levels of parasites (180 and 125 parasites/midgut respectively; Fig. 4A, *Ppap*J “no blood, d5”; Table S3). Thus, despite generation of the optimal highly substituted ‘mono-scGal’ LPG PAMP in the *Ld-cSCG3* line, survival in *Ppap*J was not enhanced (Table 1). As a control, these *Ld* transfectants were fed to *P. argentipes Parg*IN, a natural vector of *Ld* transmission originating from India [12], [24]. Previous studies have shown that midgut survival of both *L. donovani* and *L. major* in this “permissive” sand fly species is not strongly affected by LPG galactosylation patterns [8], [12], [24]. Due to the limited number of *Parg*IN flies available for analysis, a single infection time point was analyzed comparing flies without blood meal remnants in the midgut on day 5 post-feeding. In contrast to the loss of midgut infections in *Ppap*J flies, both *Ld*-*vector* and *Ld*-c*SCG3* promastigotes persisted and were maintained a moderate infection intensity in most *Parg*IN flies after the digested blood meal was expelled (88% or 78% infected flies; 11263 or 6822 parasites/midgut; Fig. 4A *Parg*IN “no blood, d5”, Table S3). These data argue against a general non-specific loss in the ability of these *Ld* transfectants to survive in the phlebotomine sand fly midgut environment.

In separate experiments *Ppap*J flies were fed on *Leishmania*-infective mouse blood containing ‘null-scGal’ *Ld*-*vector*, ‘mono-scGal’ *Ld*-p*SCG2*, or control ‘mono-scGal’ WT *L. major* FV1 promastigotes (Fig. 4B,C). As expected, most *Ppap*J flies were infected with high numbers of parasites prior to expulsion of the blood meal, although the numbers of *Ld*-*vector* and *Ld*-p*SCG2* were significantly less than control WT *L. major* FV1 (Fig. 4B “+ blood, d3” panel). However, in *Ppap*J flies that had expelled their blood meal, neither *Ld*-*vector* nor *Ld*-p*SCG2* survived (0% infected flies), whereas good survival was seen with the WT FV1 control (100% infected, 13200 parasites/midgut; Fig. 4B “no blood, d4” panel; Table S2). When *Ppap*J sand flies were infected with a 4-fold higher concentration of parasites to compensate for the diminished early growth of *Ld* transfectants compared to WT FV1, we again observed poor survival of both ‘mono-scGal’ *Ld*-p*SCG2* and ‘null-scGal’ *Ld*-*vector* parasites after the midgut blood meal had been expelled (Fig. 4C “no blood, d5” panel), despite massive parasite loads in midguts that retained their blood meals at day 3 post-feeding (Fig. 4C “+ blood, d3” panel). *Ld*-*vector* and *Ld*-p*SCG2* numbers were each significantly decreased relative to control WT FV1 (>90%, p<0.001), although a higher percentage of *Ppap*J flies remained infected (38% of *Ld-vector*, 52% of *Ld*-p*SCG2*, 100% of WT FV1; Fig. 4C “no blood, d5” panel). These results are consistent with early observations regarding the ability of high concentration of promastigotes in the artificial blood meal to overcome the natural resistance of *P. papatasi* to infection with *L. donovani* [12], [24], [46]. Together, these data suggest that while necessary for survival and transmission of *L. major* in “selective” *Ppap*J sand flies, the ‘mono-scGal’ LPG PAMP alone is not sufficient to rescue *L. donovani-SCG* promastigotes in *Ppap*J sand flies during the critical time of blood meal expulsion.

### Competition with high levels of scGal-modified secreted acid phosphatase (SAP) is unlikely to account for poor *Ppap*J survival of ‘mono-scGal’ *Ld*-*SCG* promastigotes

**Figure 5:**
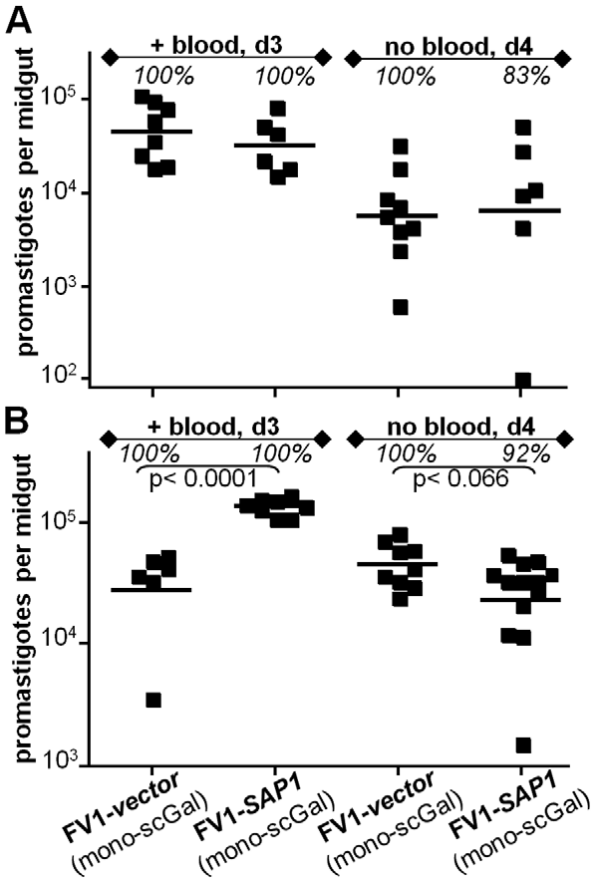
*L. major* FV1 promastigote survival in *Ppap*J infections is unaffected by over-expression of secreted acid phosphatase (SAP). Female *Ppap*J flies were membrane fed on the indicated *L. major*-infective mouse blood (4×10^6^ parasites per ml) and the number of viable parasites per midgut determined on the indicated day post-feeding as described in Fig. 1. FV1-transfectant lines are described in the text, with additional data in Table S4. High levels of active SAP were detected in the culture medium of all FV1-*SAP* transfectants, but not in WT FV1 or FV1-*vector* lines (Table S4). Results from two independent experiments (panels A,B) are shown.

Unlike *L. major*, *L. donovan*i and most other *Leishmania* species secrete high levels of acid phosphatases (SAPs) covalently modified by PG repeats [47], [48], [49]. Since PG repeats attached to SAP bear the same covalent side chain modifications as LPG PG repeats [29], [41], [50], ‘mono-scGal’ SAP could potentially compete for *Ld*-c*SCG3* and *Ld*-p*SCG2* promastigote binding to *Ppap*J midgut PpGalec receptors, thereby accounting for their failure to survive expulsion of the digested blood meal. To test this hypothesis, we engineered *L. major* FV1 to express high levels of SAP (Methods). High levels of active SAP were detected in the culture medium of all FV1-*SAP* transfectant lines, more than 1100 times higher than SAP levels in WT FV1 or control FV1-*vector* transfectant culture media and thus comparable to SAP levels secreted by *L. donovani* and other *Leishmania* species promastigotes (Table S4). However, in two independent experiments involving infections of *Ppap*J flies with *L. major*-infective mouse blood, FV1-*SAP1* over-expressors survived as well as control FV1-*vector* promastigotes, both prior to and after expulsion of the digested blood meal (Fig. 5A,B “+ blood, d3” and “no blood, d4” panels, respectively). These data argue that competition with ‘mono-scGal’ SAP is unlikely to account for the poor *Ppap*J survival of *Ld*-*SCG* transfectants expressing the ‘mono-scGal’ LPG PAMP preferred by this sand fly.

## Discussion

In this report we have studied the ability of parasites bearing different LPG side chain galactosylation PAMPS to interact with a “selective” sand fly *Leishmania* host, *Phlebotomus papatasi Ppap*J originating from the Jordan Valley. Previous data have shown that galactosylated LPG plays a key role in mediating *L. major* midgut survival and binding in this sand fly species, and that binding was mediated by the *Ppap*J midgut LPG receptor PpGalec [12], [19], [21], [24]. We show here first that this scGal-LPG PAMP is more complex than originally proposed, as only parasites bearing short ‘mono-scGal’ LPG PAMPs survived expulsion of the digested blood meal in infected *Ppap*J sand flies. This was shown using both natural *L. major* isolates exhibiting a wide range of scGal-LPG PAMPs (Fig. 1) and isogenic derivatives of the normally scGal-deficient *L. major* strain SD engineered to express different scGal-LPG PAMPs via transfection of different *SCG*-encoded PG-side chain-(β1,3)galactosyltransferases (Fig. 2). Thus, like the fairy-tale character Goldilocks, *Ppap*J sand flies show an exquisite specificity for the “just right” ‘mono-scGal’ LPG PAMP, rejecting those scGal-LPG PAMPs that are either “too short” (‘null scGal’, <0.02 avg. scGal chain length) or “too long” (‘oligo-scGal’ and ‘poly-scGal’; ≥1.9 avg. scGal chain length).

Several molecular scenarios may account for the inability of *L. major* promastigotes expressing ‘oligo-scGal’ or ‘poly-scGal’ LPG to survive expulsion of the blood meal in *Ppap*J flies. Since all *Ppap*J-competent lines expressed LPG containing a high percentage of PG repeats bearing a single βGal residue (50–59%, Table S1), it seems likely that the low level of mono-galactosylated PG repeats in ‘oligo-scGal’ and ‘poly-scGal’ *L. major* lines (6–9%, Table S1) is not sufficient to mediate binding to PpGalec midgut receptors. An alternative, non-exclusive model considers interference by modified PG repeats decorated with long chains of poly-scGal residues, which could sterically interfere with the productive binding of the mono-galactosylated PG repeats. This latter model could be probed by testing parasites bearing LPG substitutions clustered differentially along the “backbone” of polymeric PG repeats; however, methods for engineering such parasites are not yet available.

### Do scGal-LPG modifications control *L. major* “selectivity” in all *Phlebotomus papatasi*?

As noted earlier, many workers have grouped sand fly species according to their ability to support in experimental infections the survival (and, in some cases, experimental transmission) of a wide versus limited range of *Leishmania species* [7], [8], [12], [16], with the former group termed “permissive” sand flies and the latter termed “selective” or “restricted”. The availability of *Leishmania* mutants specifically defective in LPG (through the deletion of the gene encoding the LPG-specific galactofuranosyltransferase *LPG1*) has shown that in general, “selective” sand fly species show a strong role for LPG in midgut survival and binding, while the “permissive” sand fly species show little LPG dependency [7], [8], [12], [16], [17], [24]. Our panel of engineered and natural *L. major*, varying greatly in scGal-LPG modification, allowed us to compare the effects seen in a “selective” sand fly, *P. papatasi Ppap*J from the Jordan Valley, which showed a strong preference for ‘mono-scGal’ LPG PAMPs (Figs. 1–3).

Recently, we have completed studies of more than 15 *L. major* isolates that reveal a range in the extent of procyclic promastigote scGal-LPG modification, with a general cline proceeding from scGal-deficient ‘null-scGal’ LPG modification in West Africa to short chain ‘mono-scGal’ modification in the Middle East to long chain ‘poly-scGal’ modification in Central Asia (Cardoso *et al*., in preparation). Together with the findings presented here, the stage is now set for further explorations of the role of scGal-LPG PAMPs in *L. major* transmission in other natural settings. Since one natural *P. papatasi* sand fly vector in this geographic range showed differing abilities to support *Leishmania* growth which were dependent on scGal-LPG PAMPs (Figs. 1–3), it seems likely these may play an important role and perhaps even a driving force in the evolution of parasite/vector selectivity. For example, all Israeli *L. major* lines whose LPG has been characterized show ‘mono-scGal’ LPG PAMPs (V121 strain, avg. 1.1 scGal length; L580 strain, avg. 0.7 scGal length; calculated from data in [25], [27]) and correspondingly, the ability of a *P. papatasi Ppap*J colony established from wild caught flies from the Jordan Valley to support *L. major* midgut survival is strongly dependent on this scGal-LPG PAMP. In this respect it will be interesting to examine the properties of *P. papatasi* sand flies from Central Asia, including potential structural diversity in their PpGalec midgut LPG receptor, as *L. major* from this region typically elaborate a ‘poly-scGal’ LPG PAMP similar to that of LV39c5 (Cardoso *et al*., in preparation). Our work demonstrating a geographical origin-based specificity between *Ppap*J sand fly vector and *L. major* strains also complements the work of Elfari *et al.* [51] who demonstrated evidence for genetic and biological diversity in *L. major* strains that correlated with geographical origin and their ability to infect only sympatric animal reservoir hosts.

### “*Ppap*J-optimal” scGal-LPG modifications are not sufficient to confer *Ppap*J midgut survival to *L. donovani*

While expression of appropriate scGal-LPG PAMPs is necessary for the survival of *L. major* in the *Ppap*J sand fly midgut, is it sufficient? We tested this by engineering the ‘mono-scGal’ LPG PAMP into a Sudanese strain of *L. donovani* which normally expresses a completely unmodified LPG coat [45]. We showed by biochemical analyses and agglutination tests (Table S1, [30], [31]) that the engineered scGal-LPG PAMPs in *L. donovani-SCG* transfectants were faithful replicas of *L. major* ‘mono-scGal’ LPG PAMPs synthesized by natural WT *L. major* FV1 and engineered SD-*SCG3* transfectants, all of which exhibited robust long-term survival in *Ppap*J laboratory infections (Tables 1, S2). However, *L. donovani*-*SCG* lines bearing a ‘mono-scGal’ LPG surface coat remained unable to survive following expulsion of the blood meal in infected *Ppap*J flies (Fig. 4; Tables 1, S2, S3).

We then explored several possible mechanisms that could account for the failure of *L. donovani* bearing an *L. major* FV1 LPG “surface” to survive. First was the possibility that secretion of scGal-modified acid phosphatases (SAPs, [47], [48], [50]) competed for LPG-dependent midgut binding and parasite survival. While SAP-deficient *L. donovani* are not available, reconstruction experiments in *L. major* FV1 promastigotes expressing high levels of PG-modified SAPs (Fig. 5, Table S4) failed to reveal any alterations in *Ppap*J survival. Thus, competition by *L. donovani* scGal-SAP is unlikely to account for the failure of *Ld*-*SCG* promastigotes to survive in *Ppap*J midguts. A second reason was that the engineered ‘mono-scGal’ *L. donovani* were unable to withstand *Ppap*J midgut conditions, since early killing of *L. donovani* promastigotes in the *P. papatasi* midgut has been reported [52]. In fact, in comparison to the sympatric *L. major* FV1 strain, the *L. donovani* lines showed reduced growth in the early blood fed midgut (Fig. 4B), due either to their slower generation times, and/or their greater sensitivity to midgut digestive enzymes. Nevertheless, when the differences in the concentration of parasites present prior to blood meal excretion were overcome by initiating infection with a high dose inoculum, the *L. donovani* lines were still largely absent in flies that had passed their blood meals (Fig. 4C). Furthermore, *L. donovani* transfectants were able to survive within the midgut of *P. argentipes Parg*IN sand flies (Fig. 4A). Importantly, survival in this sand fly species cannot be attributed simply to a more permissive midgut environment, as *P. argentipes* restricts survival of *lpg2*- *Ld* lines which lack LPG and other PGs, evidence of a strongly hydrolytic midgut environment [8], [24]. These data argue that the inability of WT or engineered *L. donovani* lines to survive in *Ppap*J sand flies is not due to an inability to withstand the midgut environment, and the timing of the loss of infection is consistent with their failure to attach to the midgut.

### Additional factors may be required to mediate *Leishmania* - sand fly midgut interactions

Thus, while specific scGal-LPG PAMPs are necessary for *L. major* persistence and midgut binding during expulsion of the blood meal in *Ppap*J flies, the inability of *L. donovani* expressing the appropriate *L. major* scGal-LPG PAMP to survive in the same fly strain suggests most simply that this interaction, while necessary, is not sufficient for midgut attachment. This in turn would argue that an additional parasite ligand(s) must be required, one shared in the closely related *L. major* strains but lacking in *L. donovani*, which diverged from *L. major* >80 million years ago [53]. In this model, generation of proper scGal-LPG PAMPs in *L. major* SD would be sufficient to promote survival, since *L. major* strains would retain this second *L. major*-specific interaction; but insufficient in *L. donovani*, where the second interaction was absent due to evolutionary divergence or loss. In contrast to *Ld-SCG* transfectants, which “inherited by transfection” only the scGal-LPG-dependent ligand, the enhanced *P. papatasi* survival of *L. infantum* - *L. major* hybrids observed by Volf *et al.* (relative to *L. infantum*; [54]) is thus predicted to result from the inheritance of both *L. major*-specific scGal-LPG-dependent and -independent ligands.

Whether this postulated second interaction is mediated through a second species-specific receptor for LPG, or an LPG-independent ligand such as the one proposed by Myskova *et al.* to control midgut binding of certain *Leishmania* species in “permissive” sand fly vectors [8], [16], is unknown. Perhaps the *Ppap*J ‘mono-scGal’ LPG midgut receptor PpGalec collaborates with a co-receptor, similar to the interactions of certain other pattern recognition receptors such as Toll-like receptors (TLR1/2/6) with each other or with other receptors (Dectin-1, CD14, TLR4; reviewed in [55], [56].

This putative species-specific co-receptor may be especially relevant to the interaction of *L. major* strains with *P. duboscqi* sand flies. This vector, while unable to support the survival of *L. major* lines completely deficient in LPG biosynthesis [8], [22], [44], is not sensitive to differences in *L. major* LPG galactosylation patterns (Fig. S1) and naturally transmits *L. major* strains in West Africa bearing effectively ‘null-scGal’ LPG. Nonetheless, *P. duboscqi* is a “selective” vector, permitting only the development of *L. major* in experimental infections ([8], Sacks *et al*., unpublished). These data suggest that the few interactions between predicted *P. duboscqi* PpGalec midgut LPG receptors [19] and the low number of mono-galactosylated PG repeats in WT SD LPG (2%, Table S1) is sufficient to mediate parasite attachment to the *Pdub*M midgut epithelium, in concert with a second *L. major*-specific midgut binding interaction that is especially strong in this particular sand fly species. It is also possible that a scGal-independent ligand present on *L. major* LPG binds to the alternative receptor and is a sufficient interaction to maintain infection in the *Pdub*M vector. When the factor(s) controlling parasite LPG-independent binding and survival of *Leishmania* in “selective” and “permissive” sand fly species becomes known, it should be possible to test these hypotheses.

## Supporting Information

Figure S1 Survival of natural and isogenic lines of *L. major* in *P. duboscqi*, the natural vector of *L. major* in West Africa, is independent of scGal-LPG PAMPs. Female *P. duboscqi* sand flies originating from Mali (*Pdub*M) were fed on the indicated *L. major*-infective mouse blood and the number of viable parasites per midgut determined on the indicated day post-feeding, as described in Fig. 1. SD-transfectant lines are described in the text, with additional data in Fig. 2 and Table S1. Infective mouse blood contained 5x 10^6^ (panel A) or 4 x 10^6^ (panel B) parasites per ml. Results from two independent experiments are shown.
(0.32 MB TIF)

Table S1 scGal-LPG profiles of *Leishmania* lines used in this study.
(0.33 MB DOC)

Table S2 *P. papatasi Ppap*J sand fly infection outcomes after expulsion of the digested blood meal.
(0.06 MB DOC)

Table S3 Comparative outcomes in *P. papatasi Ppap*J and *P. argentipes Parg*IN infections after expulsion of the digested blood meal.
(0.03 MB DOC)

Table S4 Secreted acid phosphatase (SAP) levels in *Leishmania* procyclic promastigote culture medium.
(0.03 MB DOC)
