# Supplementary figures and images for: Leishmania major Survival in Selective Phlebotomus papatasi Sand Fly Vector Requires a Specific SCG-Encoded Lipophosphoglycan Galactosylation Pattern
Source: PLoS Pathog. 2010 Nov 11;6(11):e1001185. doi: 10.1371/journal.ppat.1001185 (PMC2978724; doi:10.1371/journal.ppat.1001185)

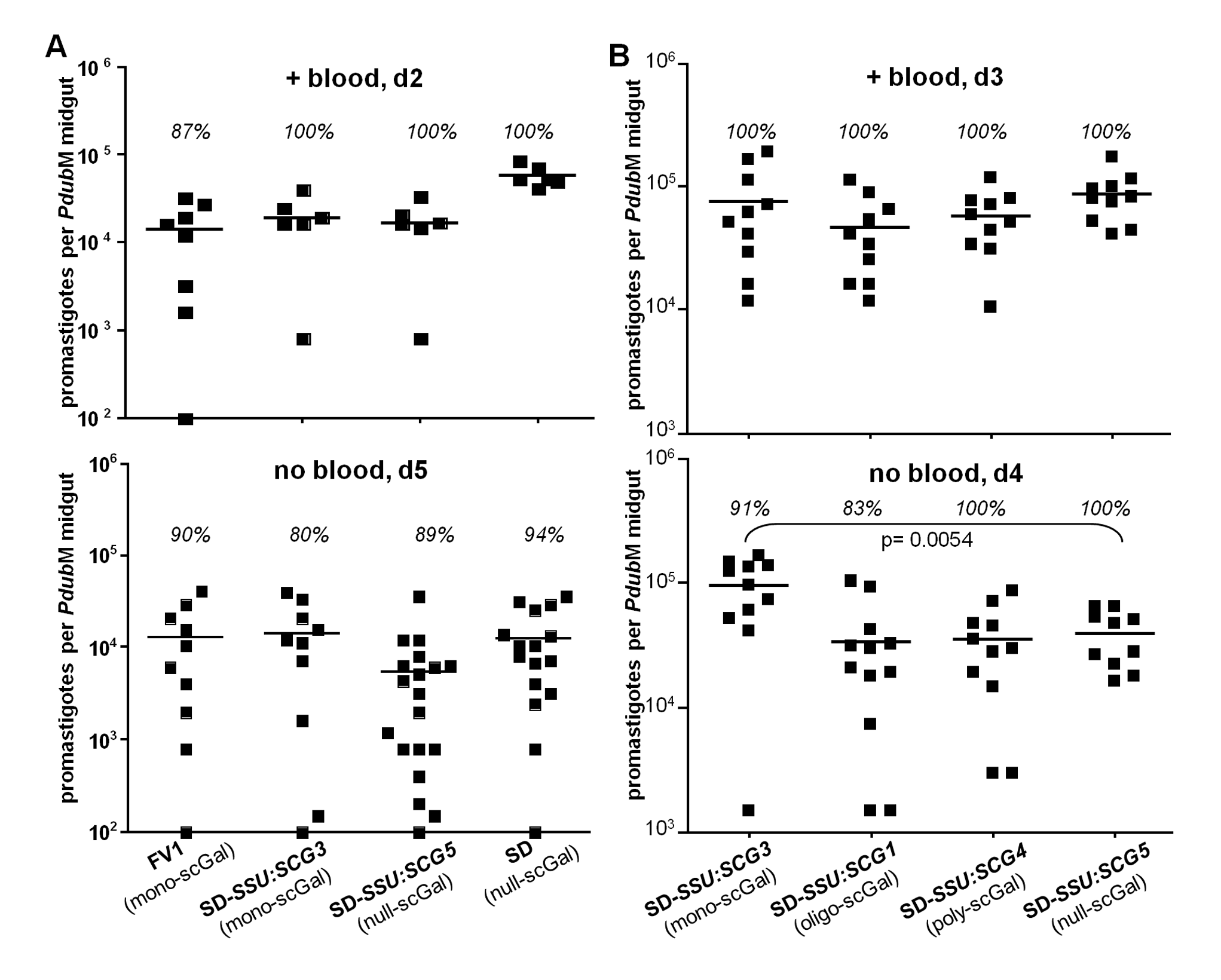

Supplement: Figure S1 — Survival of natural and isogenic lines of L. major in P. duboscqi, the natural vector of L. major in West Africa, is independent of scGal-LPG PAMPs. Female P. duboscqi sand flies originating from Mali (PdubM) were fed on the indicated L. major-infective mouse blood and the number of viable parasites per midgut determined on the indicated day post-feeding, as described in Fig. 1. SD-transfectant lines are described in the text, with additional data in Fig. 2 and Table S1. Infective mouse blood contained 5x 10^6^ (panel A) or 4 x 10^6^ (panel B) parasites per ml. Results from two independent experiments are shown. (0.32 MB TIF) [file ppat.1001185.s001.tif]
