# Supplementary material for: Leishmania major Survival in Selective Phlebotomus papatasi Sand Fly Vector Requires a Specific SCG-Encoded Lipophosphoglycan Galactosylation Pattern
Source: PLoS Pathog. 2010 Nov 11;6(11):e1001185. doi: 10.1371/journal.ppat.1001185 (PMC2978724; doi:10.1371/journal.ppat.1001185)
Supplement: Table S1 — scGal-LPG profiles of Leishmania lines used in this study. (0.33 MB DOC) [file ppat.1001185.s002.doc]

| ***Leishmania* lines^a^** | **construct^b^** | **% LPG repeats with indicated terminal scGal length^c^** | | | | | | | | | | | | | **mean scGal chain length^d^** | **LPG-scGal frequency^e^** | **avg. scGal chain length^f^** |
| --- | --- | --- | --- | --- | --- | --- | --- | --- | --- | --- | --- | --- | --- | --- | --- | --- | --- |
|  |  | **0** | **1** | **2** | **3** | **4** | **5** | **6** | **7** | **8** | **9** | **10** | **11** | **12** |  |  |  |
| WT FV1 |  | 21 | 59 | 11 | 1 |  |  |  |  |  |  |  |  |  | 1.2 | 71 | 0.8 |
| WT LV39c5 |  | 7 | 16 | 22 | 16 | 17 | 11 | 7 | 3 | 2 |  |  |  |  | 3.3 | 93 | 3.1 |
| WT SD |  | 98 | 2 |  |  |  |  |  |  |  |  |  |  |  | 1.0 | 2 | 0.02 |
| SD-*SSU:SCG5* | B5170, *SSU*:IR1sat *SCG5* | 98 | 2 |  |  |  |  |  |  |  |  |  |  |  | 1 | 2 | 0.02 |
| SD-c*SCG3* | B3979, cosmid *SCG3* | 28 | 53 | 6 | 9 |  |  |  |  |  |  |  |  |  | 1.4 | 68 | 0.9 |
| SD-*SSU:SCG3* | B5101, *SSU*:IR1sat *SCG3* | 10 | 49 | 30 | 7 |  |  |  |  |  |  |  |  |  | 1.5 | 86 | 1.3 |
| SD-*SSU:SCG1* | B5097, *SSU*:IR1sat *SCG1* | 33 | 17 | 8 | 6 | 6 | 7 | 7 | 5 |  |  |  |  |  | 3.3 | 56 | 1.9 |
| SD-*SSU:SCG4* | B5103,  *SSU*:IR1sat *SCG4* | 39 | 6 | 4 | 4 | 5 | 6 | 6 | 6 | 6 | 4 | 3 | 2 | 2 | 5.8 | 54 | 3.1 |
| *Ld*-*vector* | B890, c*LHYG* vector | 100 |  |  |  |  |  |  |  |  |  |  |  |  | 0 | 0 | 0 |
| *Ld*-c*SCG3* | B3979, cosmid *SCG3* | 31 | 63 | 2 |  |  |  |  |  |  |  |  |  |  | 1.0 | 65 | 0.7 |
| *Ld* -p*SCG2* | B3900, pXK-*SCG2* | 17 | 25 | 26 | 11 |  |  |  |  |  |  |  |  |  | 1.8 | 62 | 1.1 |

**^a^**The *L. major* (FV1, LV39c5, SD) and *L. donovani* (*Ld*) wild-type (“WT”) and transfectant lines used in these studies are described in the text.

**^b^**For each *Leishmania* transfectant, the *Leishmania* episomal contruct SMB laboratory identification number (“Bxx”) is followed by a brief description; see Methods for detailed construct information.

**^c^**The percentage of purified procyclic promastigote LPG PG repeats bearing terminal βGal side chains with the indicated number of residues (“scGal length”) was rounded to the nearest whole integer. “0” denotes unmodified PG repeats with no side chain sugars. Modified PG repeats bearing arabinose-capped βGal side chains were excluded from this Table.

**^d^**The mean number of scGal residues per purified LPG repeat, or “mean scGal chain length”, was calculated by adding together the product of [(number of βGal residues per side chain) x (% PG repeats with that terminal scGal length)] for every terminal scGal-modified LPG repeat species, then dividing the sum by the total percentage of terminal scGal-modified LPG repeats (“LPG-scGal frequency”).

**^e^**“LPG-scGal frequency” is the total percentage of terminal scGal-modified PG repeats observed in purified procyclic promastigote LPG samples.

**^f^**The average number of scGal residues per purified LPG PG repeat, or “avg. scGal chain length”, was calculated as the product of (“mean scGal chain length”) x (“LPG-scGal frequency”).
