# Supplementary material for: Leishmania major Survival in Selective Phlebotomus papatasi Sand Fly Vector Requires a Specific SCG-Encoded Lipophosphoglycan Galactosylation Pattern
Source: PLoS Pathog. 2010 Nov 11;6(11):e1001185. doi: 10.1371/journal.ppat.1001185 (PMC2978724; doi:10.1371/journal.ppat.1001185)
Supplement: Table S2 — P. papatasi PpapJ sand fly infection outcomes after expulsion of the digested blood meal. (0.06 MB DOC) [file ppat.1001185.s003.doc]

| ***Leishmania* line^a^** | ***Ppap*J infection I** | | | | ***Ppap*J infection II** | | | | **average *Ppap*J survival^f^** |
| --- | --- | --- | --- | --- | --- | --- | --- | --- | --- |
|  | **% infected flies^b^** | **mean parasites/midgut^c^** | **relative parasites/midgut^d^** | **relative survival^e^** | **% infected flies** | **mean parasites/ midgut** | **relative parasites/ midgut** | **relative survival** |  |
| WT FV1 | 94 | 23720 | 100 | 94 | 82 | 16212 | 100 | 82 | 88 + 6 |
| WT LV39c5 | 69 | 9566 | 40 | 28 | 53 | 3861 | 24 | 13 | 20 + 8 |
| WT SD |  | | | | 38 | 6660 | 41 | 16 | 16* |
|  |  | | | |  | | | |  |
| WT FV1 | 100 | 13500 | 100 | 100 | 100 | 30000 | 100 | 100 | 100 |
| SD-c*SCG3* | 86 | 18600 | 138 | 118 | 94 | 18890 | 63 | 59 | 89 + 30 |
| SD-*SSU:SCG3* | 76 | 18750 | 139 | 106 | 100 | 13160 | 44 | 44 | 75 + 31 |
| SD-*SSU:SCG1* | 55 | 4116 | 30 | 17 | 37 | 12510 | 42 | 15 | 16 + 1 |
| SD-*SSU:SCG4* | 56 | 683 | 5 | 3 | 42 | 2267 | 8 | 3 | 3 + 1 |
| SD-*SSU:SCG5* | 62 | 2563 | 19 | 12 | 60 | 2415 | 8 | 5 | 8 + 3 |
|  |  | | | |  | | | |  |
| *Ld-vector* | 38 | 4500 | 8 | 3 | 0 | 0 | 0 | 0 | 1 + 1 |
| *Ld*-p*SCG2* | 52 | 5000 | 8 | 4 | 0 | 0 | 0 | 0 | 2 + 2 |
| WT FV1 | 100 | 59000 | 100 | 100 | 100 | 13200 | 100 | 100 | 100 |

**^a^**The *L. major* (FV1, LV39c5, SD) and *L. donovani* (*Ld*) WT and transfectant lines used in six independent *P. papatasi* *Ppap*J laboratory infections are described in the text and Table S1.

**^b^**“% infected flies” is the percentage of sand flies retaining infections after expulsion of the digested blood meal, using data presented in Figs. 1, 2, 4B, and 4C. In each experiment, a total of 8-21 midguts were analyzed for each parasite line.

**^c^**The mean number of parasites per midgut post-blood meal expulsion, or “mean parasites/midgut”, was calculated using data presented in Figs. 1, 2, 4B, and 4C.

**^d^**“Relative parasites/midgut” is the mean number of parasites per midgut post-blood meal expulsion calculated relative to control WT FV1 = 100.

**^e^**“Relative survival” of parasites post-blood meal expulsion was calculated by multiplying (“% infected flies”) x (“relative parasites per midgut”).

**^f^**“Average *Ppap*J survival” is the average (+ SEM) relative parasite survival in 2 or more independent *Ppap*J infections; “*” denotes data from a single experiment.
