# Supplementary material for: Leishmania major Survival in Selective Phlebotomus papatasi Sand Fly Vector Requires a Specific SCG-Encoded Lipophosphoglycan Galactosylation Pattern
Source: PLoS Pathog. 2010 Nov 11;6(11):e1001185. doi: 10.1371/journal.ppat.1001185 (PMC2978724; doi:10.1371/journal.ppat.1001185)
Supplement: Table S3 — Comparative outcomes in P. papatasi PpapJ and P. argentipes PargIN infections after expulsion of the digested blood meal. (0.03 MB DOC) [file ppat.1001185.s004.doc]

| ***Leishmania* line^a^** | ***Ppap*J infection^b^** | | | | ***Parg*IN infection^b^** | | | |
| --- | --- | --- | --- | --- | --- | --- | --- | --- |
|  | **% infected flies** | **mean parasites/midgut** | **relative parasites/midgut** | **relative survival** | **% infected flies** | **mean parasites/midgut** | **relative parasites/midgut** | **relative survival** |
| *Ld*-*vector* | 9 | 180 | 3^ | 0.3^ | 88 | 6860 | 100 | 88 |
| *Ld*-c*SCG3* | 8 | 125 | 2^ | 0.2^ | 77 | 11400 | 166 | 128 |

**^a^***L. donovani* (*Ld*) lines used in laboratory infections of *P. papatasi* *Ppap*J (“*Ppap*J”) and *P. argentipes* *Parg*IN (“*Parg*IN”) sand flies are described in the text and Table S1.

**^b^**The percentage of sand flies retaining infection after expulsion of the digested blood meal (“% infected flies”), and the mean number of parasites per midgut post-blood meal expulsion (“mean parasites/midgut”), relative number of parasites per midgut post-blood meal expulsion (“relative parasites/midgut”), and relative parasite survival post-blood meal expulsion (“relative survival”) were determined as described in Table S2, using data presented in Fig. 4A. ^Since WT FV1 was not included in this experiment, and *P. argentipes* *Parg*IN flies are the natural vector of *Ld* parasites, calculations were relative to *Ld*-*vector*-infected *Parg*IN flies = 100.
