# Supplementary material for: Leishmania major Survival in Selective Phlebotomus papatasi Sand Fly Vector Requires a Specific SCG-Encoded Lipophosphoglycan Galactosylation Pattern
Source: PLoS Pathog. 2010 Nov 11;6(11):e1001185. doi: 10.1371/journal.ppat.1001185 (PMC2978724; doi:10.1371/journal.ppat.1001185)
Supplement: Table S4 — Secreted acid phosphatase (SAP) levels in Leishmania procyclic promastigote culture medium. (0.03 MB DOC) [file ppat.1001185.s005.doc]

| **source^a^** | **SAP^b^** | **relative SAP^c^** |
| --- | --- | --- |
| WT FV1 | 377 | 1 |
| FV1- *vector* | 164 | 0 |
| FV1-*SAP1* | 419987 | 1114 |
| FV1- *SAP2* | 456623 | 1211 |
| WT *L. mexicana* | 945458 | 2508 |

**^a^** *L. major* FV1 and *L. mexicana* wild-type (“WT”) and transfectant lines from which culture supernatants were obtained are described in Methods. *L. mexicana*, which secretes high levels of SAP comparable to *Ld* [49], was included as a positive control in these experiments.

**^b^**The amount of active SAP present in culture supernatants was determined as described (Methods), using non-denaturing polyacrylamide gel electrophoresis and an *in situ* SAP enzyme activity stain. Background-corrected SAP levels are reported as integrated SAP density units in 10 microliter culture supernatant samples.

**^c^**“Relative SAP” levels were calculated relative to WT FV1 SAP = 1.
